# Feasibility and effects of patient-cooperative robot-aided gait training applied in a 4-week pilot trial

**DOI:** 10.1186/1743-0003-9-31

**Published:** 2012-05-31

**Authors:** Alex Schück, Rob Labruyère, Heike Vallery, Robert Riener, Alexander Duschau-Wicke

**Affiliations:** 1 Sensory-Motor Systems Lab, Institute of Robotics and Intelligent Systems, Department of Mechanical and Process Engineering,, ETH Zurich, Zurich, Switzerland; 2 Spinal Cord Injury Center, University Hospital Balgrist, University of Zurich, Zurich, Switzerland; 3 Institute of Human Movement Sciences and Sport, ETH Zurich, Zurich, Switzerland; 4, University College Physiotherapy Thim van der Laan, Landquart, Switzerland; 5, Hocoma AG, Volketswil, Switzerland; 6 Biomedical Engineering, Khalifa University of Science, Technology & Research, Abu Dhabi, UAE

## Abstract

**Background:**

Functional training is becoming the state-of-the-art therapy approach for rehabilitation of individuals after stroke and spinal cord injury. Robot-aided treadmill training reduces personnel effort, especially when treating severely affected patients. Improving rehabilitation robots towards more patient-cooperative behavior may further increase the effects of robot-aided training. This pilot study aims at investigating the feasibility of applying patient-cooperative robot-aided gait rehabilitation to stroke and incomplete spinal cord injury during a therapy period of four weeks. Short-term effects within one training session as well as the effects of the training on walking function are evaluated.

**Methods:**

Two individuals with chronic incomplete spinal cord injury and two with chronic stroke trained with the Lokomat gait rehabilitation robot which was operated in a new, patient-cooperative mode for a period of four weeks with four training sessions of 45 min per week. At baseline, after two and after four weeks, walking function was assessed with the ten meter walking test. Additionally, muscle activity of the major leg muscles, heart rate and the Borg scale were measured under different walking conditions including a non-cooperative position control mode to investigate the short-term effects of patient-cooperative versus non-cooperative robot-aided gait training.

**Results:**

Patient-cooperative robot-aided gait training was tolerated well by all subjects and performed without difficulties. The subjects trained more actively and with more physiological muscle activity than in a non-cooperative position-control mode. One subject showed a significant and relevant increase of gait speed after the therapy, the three remaining subjects did not show significant changes.

**Conclusions:**

Patient-cooperative robot-aided gait training is feasible in clinical practice and overcomes the main points of criticism against robot-aided gait training: It enables patients to train in an active, variable and more natural way. The limited number of subjects in this pilot trial does not permit valid conclusions on the effect of patient-cooperative robot-aided gait training on walking function. A large, possibly multi-center randomized controlled clinical trial is required to shed more light on this question.

## Background

Patients with motor dysfunction due to lesions of their central nervous system (CNS) typically undergo physical and occupational therapy for rehabilitation. In the past, this therapy mainly consisted of stretching, bracing and strengthening of the affected limbs as well as training of compensation strategies using unaffected limbs to allow patients to become as functional and independent as possible [1]. Since research on neural plasticity has started to stress the ability of the CNS to reorganize and relearn, therapy approaches have emerged that focus on exploiting this plasticity in a functionally beneficial way. A prominent approach in this direction has been pioneered by the American psychologist Edward Taub. Based on the psychological concept of "learned helplessness" [2], he argued that especially hemiplegic patients may end up in a state of "learned non-use" of their paretic limbs if they are not sufficiently encouraged to use them. This (psychological) state would then also be unfavorable in the perspective of neural plasticity, as the CNS is not driven to reorganize itself in support of the affected limbs. To prevent these negative developments, Taub and his colleages designed the technique of constraint-induced movement therapy (CIMT), also referred to as "forced use" or "constrained-induced therapy" [3], which has shown to be effective in randomized controlled trials [4-6]. Furthermore, animal studies are starting to shed light on the underlying physiological mechanisms of recovery induced by CIMT [7].

### Body-weight supported treadmill training

In the light of neural plasticity and basic principles of motor learning it seems apparent that rehabilitation training should be task specific, i.e. if the aim is to relearn walking, one should practice walking. This common-sense argument for task specificity has been demonstrated to be valid by a large body of research, e.g. [8-12].

To perform task-specific gait training in a safe environment, body-weight supported treadmill training (BWSTT) has been introduced [13,14]. In this approach, patients wear a harness and are partially relieved from their body weight by a body-weight support system. BWSTT has become a well-accepted task-specific therapy to retrain gait in individuals with neurologically caused walking impairments. While BWSTT does not appear to be more effective than other task-specific approaches such as overground mobility training [15,16], it causes better results than unspecific lower extremity strength training [17].

### Robot-assisted gait training

For severely affected patients, task-specific training for walking—be it on a treadmill or overground—puts a substantial physical burden on therapists, who not only have to facilitate the desired movements of paretic and potentially spastic limbs but also need to guarantee the safety of their patients and prevent them from falling. Therefore, even with the additional safety of the body-weight support system in BWSTT, two or three therapists are needed to train a para- or tetraplegic individual.

These limitations motivated interdisciplinary teams of engineers and clinicians to develop technical tools that reduce the burden of manually assisted gait training. In the late 90s of the 20th century, the Lokomat [18,19] and the gait trainer GT1 [20] were developed and subsequently commercialized.

Both devices became commercially successful and were soon followed by others: The AutoAmbulator/ReoAmbulator (Motorika) [21], and the Haptic Walker [22], which has been commercialized as G-EO (RehaTechnologies^a^).

In addition to the commercially available gait rehabilitation robots, countless research prototypes have been developed, e.g. [23-26].

Clinical research about the efficacy of robot-aided gait training is still at an early, rather inconclusive state [27,28]. For the Lokomat, studies with stronger focus on non-ambulatory subjects found advantages of robot-aided gait training over manually assisted gait training [29-32], while studies focusing on ambulatory subjects found manually assisted gait training to be more effective [33,34]. These results suggest that currently, robot-aided treadmill training is most effective for severely affected, non-ambulatory patients, whereas it may not be ideal for more advanced, ambulatory patients. This situation demonstrates the need to improve current rehabilitation robots in a way that extends their spectrum of effective treatment to functionally more advanced patients. Such an improvement would allow patients to benefit from robot-aided treadmill training throughout their different stages of recovery, up to a point where they can safely and efficiently perform overground training.

Furthermore, the repetitive gait pattern imposed by a position-controlled robot may not provide sufficient variability to drive the reorganization of the CNS in an optimal way. Variability in the input data is required for neural networks to improve fault-tolerance, generalization, and learning [35]. In training theory, this observation has been well-known for a long time, and it has been captured nicely in the phrase coined by Bernstein that training should be "repetition without repetition" [36].

### Previous work

The first controllers for robotic devices supporting gait training were position controllers with the aim to ensure that the robot (and the patient) followed the desired movement trajectory as closely as possible [18,20]. These controllers were essentially "blind" to the actions of patients, i.e. active movements in the desired direction were equally prohibited as active movements in other directions. Thus, they did not encourage patients to participate actively in the training and reduced movement variability to a minimum.

Efforts were then taken to introduce more compliant control schemes, particularly *impedance control*[37]. Here, the current position of the robot is virtually coupled to a reference position by a simulated spring and damper assembly with adjustable stiffness and damping values. The spring and damper action is emulated by motors, which apply forces on the patient. With reduced spring stiffness, patients can participate more actively and experience more movement variability [38]. However, they can also lead to unfavorable movement patterns and become more and more affected by the inertia of the robots as impedance is reduced.

The *assist-as-needed (AAN) paradigm*[39,40] has been introduced to generally improve the behavior of rehabilitation robots. It states that a rehabilitation robot should constantly try to reduce its support so that patients receive just the minimum of support required to overcome their weakness or impaired motor control. Then, patients would not be able to "slack" and rely on the support of the robot, and the danger of "learned helplessness" would be avoided.

Besides these general trends, there have been many efforts to develop controllers aiming to improve robot-aided gait rehabilitation in recent years [24,39,41-48].

One particular approach, motivated by the experiments performed in animal experiments by Cai and colleagues [11,49], is the patient-cooperative Path Control strategy, which we implemented for the Lokomat gait rehabilitation robot. The control strategy allows patients to influence the timing of their leg movements along the spatial *path* of a physiologically meaningful walking pattern. The robot simulates compliant virtual walls, which keep the patient’s legs within a "tunnel" around the desired spatial path [50].

We have previously investigated the immediate effects of the Path Control strategy versus non-cooperative robot-aided gait training on individuals with incomplete spinal cord injury (iSCI). Eleven individuals with iSCI participated in a single training session with the Lokomat. The participants trained more actively and with larger kinematic variability while the Lokomat was controlled with the patient-cooperative Path Control strategy than during standard position-controlled robot-aided gait training [51].

### Research questions

Based on the encouraging results of the single training session with iSCI subjects, we were interested in the feasibility and potential effects of applying the patient-cooperative training approach over several weeks. Therefore, we applied this kind of training in a pilot trial on a single case basis.

The aim of this pilot trial was to answer the following research questions: 

1. Is it feasible to apply patient-cooperative robot-aided gait training over the course of a typical rehabilitation program of four weeks?

2. What is the short-term effect of patient-cooperative vs. non-cooperative robot-aided gait training on patients?

3. What is the effect of four weeks of patient-cooperative robot-aided gait training on walking function?

## Methods

### Rehabilitation device

**Figure 1:**
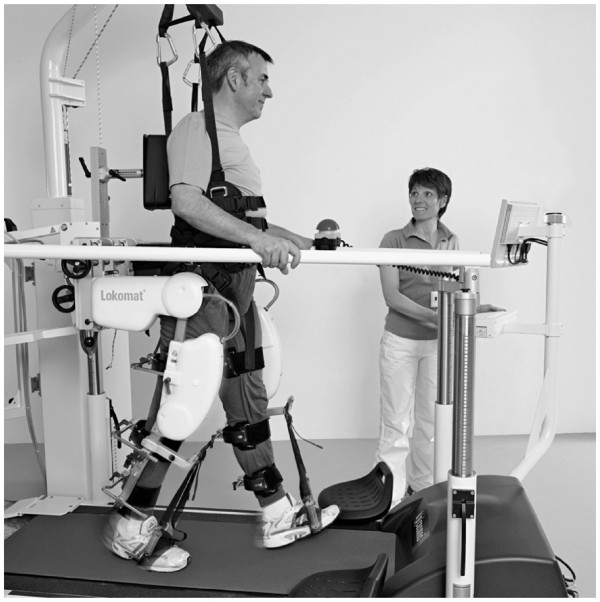
The Lokomat gait rehabilitation robot (Photo courtesy of Hocoma AG).

In this study, patient-cooperative robot-aided gait training was implemented with the Lokomat gait rehabilitation robot (Figure 1). More detailed information about the device can be found in the related publications [18,19]. Briefly summarized, the Lokomat comprises two actuated leg orthoses that are attached to the patient’s legs. Each orthosis has one linear drive in the hip joint and one in the knee joint to induce flexion and extension movements of hip and knee in the sagittal plane. Knee and hip joint torques can be determined from force sensors between actuators and orthosis. Passive foot lifters can be added to induce ankle dorsiflexion during swing phase. A body weight support system relieves patients from a definable amount of their body weight via a harness [52].

### Robotic control strategies

The main control strategy used in this trial was Generalized Elastic Path Control as introduced in [53]. In this approach, a "virtual tunnel" for the leg movements is represented by a virtual force field, represented by a mapping from the current position to the force reponse from the virtual elastic tunnel walls. The force field was obtained by an optimization algorithm, under the constraint that the field has to be conservative to ensure controller stability. Details of the procedure are reported in [53,54]. In earlier studies, the "virtual tunnel" had been implemented by means of a nearest-neighbor search with respect to a reference trajectory [50,51]. The transition from this earlier approach to the Generalized Elastic Path Control approach was made because the latter provided better control performance with more natural movements within the "virtual tunnel" [53,54].

We implemented three different conservative force fields: (1) a narrow tunnel with tight coupling between the joints, (2) a tunnel of medium width, and (3) a wide tunnel, which provided only very loose coupling. The therapist was able to continuously adjust the tunnel width between these settings, causing the control algorithm to linearly interpolate between the different force fields.

**Figure 2:**
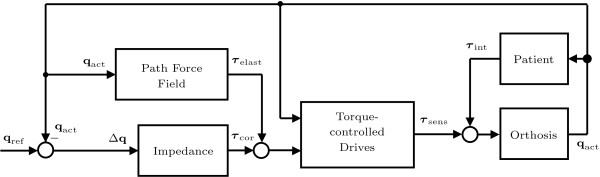
**Control scheme of the Generalized Elastic Path Control combined with the conventional impedance control approach.** The "Path Force Field" block constitutes a conservative force field and computes the torques *τ*_elast_ as a function of the actual joint angles q_act_[53]. The corrective torques *τ*_cor_ of the "Impedance" block simulate a viscoelastic coupling of the actual angles q_act_ to the reference angles q_ref_. The "torque-controlled drives" apply the desired torques to the Lokomat orthosis ("Orthosis"). These torques are measured as *τ*_sens_. Together with the interaction torques *τ*_int_, they move the exoskeleton and determine the actual angles q_act_.

We combined the Generalized Elastic Path Control approach with a standard impedance controller [38], because it was easier for patients to start the training with the reference timing provided by this strategy (Figure 2). The stiffness of this superimposed secondary controller was continuously adjustable between zero and the maximally achievable stiffness. The maximal stiffness will also be referred to as 100% guidance force, and zero stiffness as 0% guidance force. At 0% guidance force, only the virtual tunnel (and optional supportive flow) rendered by the Generalized Elastic Path Control algorithm affected the patient.

For more advanced patients, we included the possibility to influence the treadmill speed by combining the patient-cooperative Lokomat controller with the Automatic Treadmill Speed Adaptation algorithm [55]. In this approach, the horizontal ground reaction forces between the patient’s feet and the treadmill are used to intuitively control the treadmill speed during robot-aided gait training. The maximal treadmill speed for this trial was limited to 4.0 km/h (1.1 m/s).

The body-weight support system was controlled as described in [52], to provide a constant level of body-weight support set by the therapist.

**Figure 3:**
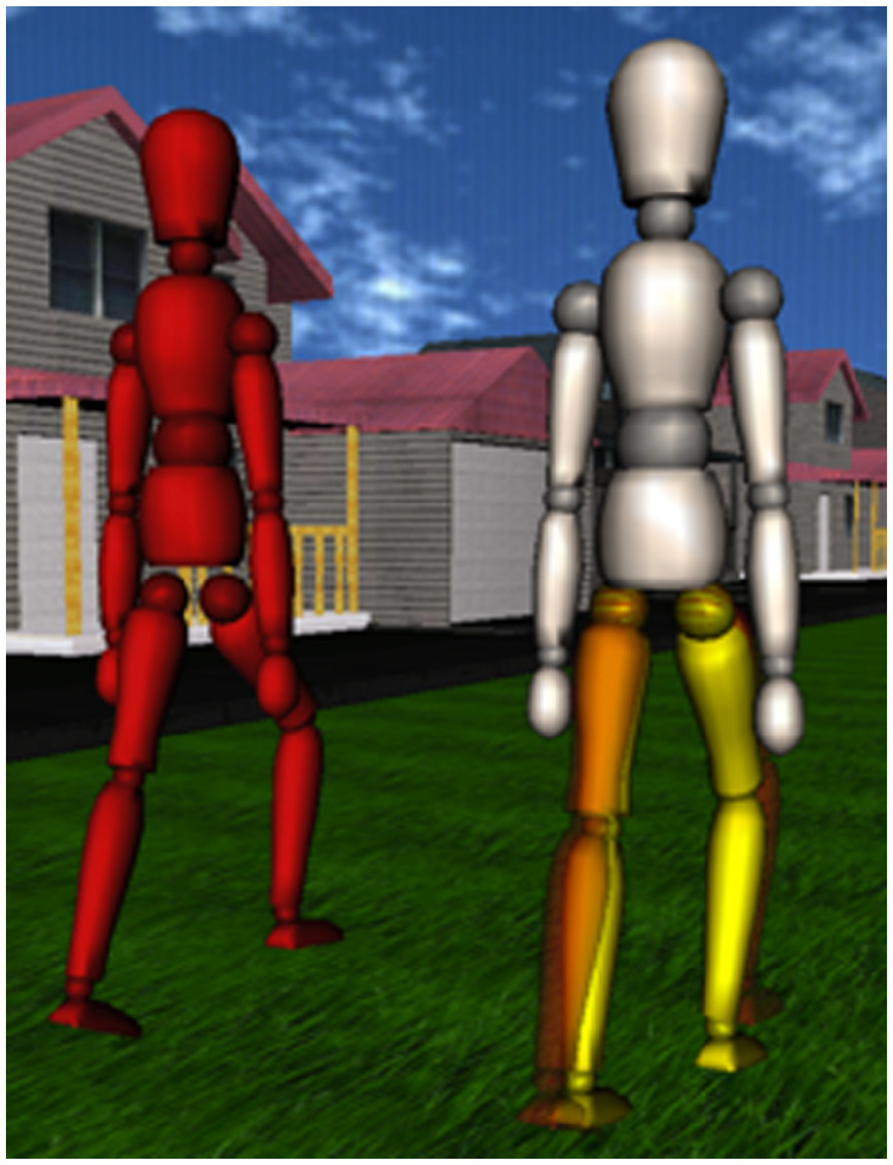
**Visual feedback for the pilot trial.** The yellow legs of the patient avatar (figure on the right) represent the actual movements of the Lokomat legs. The red figure on the left demonstrates the desired leg movements. Subjects are instructed to match the movements of the red figure as close as possible. For fine-tuning of the movements, subjects can focus on the semi-transparent red "ghost legs" that are overlaid to the legs of the patient avatar.

A similar approach as described in [50] was used for visual feedback: An avatar representing the patient was shown with overlaid "ghost legs" which demonstrated the desired movements. Additionally, a second manikin, which walked to the left of the patient avatar, was introduced. Like the "ghost legs", this second manikin showed the desired leg movements based on the original gait trajectory enclosed by the "virtual tunnel" rendered by the Generalized Elastic Path Control, with a timing appropriate for the selected treadmill speed. The patient was instructed to try to match the movements of the red manikin (Figure 3).

During training in Automatic Treadmill Speed Adaptation mode, the visual feedback was modified. The "ghost legs" were removed from the patient avatar, and the red manikin walked with a definable speed in the virtual environment. The speed of the patient avatar was coupled to the current treadmill speed, and the patient was instructed to match the speed of the red manikin. By employing a pseudo-random profile of desired speeds for the red manikin, the therapist was able to trigger patients to autonomously vary their walking speed.

### Trial location and subjects

The trial was conducted at the spinal cord injury (SCI) research lab of the Spinal Cord Injury Center at Balgrist University Hospital, Zurich, Switzerland. The study protocol was approved by the Ethics Committee of the Canton of Zurich, Switzerland, and all subjects gave written informed consent prior to the experiments.

To be considered for inclusion, iSCI subjects had to be between the age of 18 and 70 with a chronic incomplete spinal cord injury (time after injury greater than 12 months). Subjects had to be rated as ASIA C or D on the American Spinal Cord Injury Association Impairment Scale (AIS) with a motor level of lesion between C4 and Th11 [56]. Furthermore, they had to be unable to walk without at least moderate assistance at the time of inclusion (i.e. a score of less than six in the "mobility outdoors" item of the latest version of the Spinal Cord Independence Measure (SCIM III) [57] was required). Cognitive capacity to follow simple verbal instructions was necessary.

For stroke subjects, the inclusion criteria were: age between 18 and 70 years, a hemiparesis which had persisted for more than 6 months after one (but not more than one) unilateral, supratentorial, ischemic or hemorrhagic stroke without bilateral, brain stem or cerebellar lesions. The lesion had to be confirmed by radiologic findings. Subjects had to be able to ambulate more than 10 m overground without assistance of a therapist at speeds between 0.1 and 0.8 m/s, using assistive devices or braces as needed. Stroke subjects had to have at least one key muscle of the affected leg with values 3 or lower according to manual muscle testing [58,59]. Cognitive capacity to follow simple verbal instructions was necessary and was verified with the Mini-Mental State Examination [60].

Exclusion criteria for both groups were: The subject was not ambulating prior to stroke or SCI, or met one or more of the standard exclusion criteria for Lokomat training (body weight greater than 130 kg, body height greater than 2 m, leg length difference greater than 2 cm, osteoporosis, instable fracture in lower extremity, restricted range of motion, presence of decubitus ulcer of lower extremity). Also, any of the following obstructive diseases limiting training led to exclusion of the study: arthritis causing pain while stepping; dyspnea or angina on moderate exertion; limited walking endurance due to cardiopulmonary or other diseases. Prevalence of other neurological or orthopedic injuries and medical diseases which may limit exercise participation or impair locomotion (e.g. serious infection; severe orthostatic hypotension or uncontrolled hypertension, congestive heart failure, pain while weight-bearing) as well as severe metabolic diseases, epilepsy, pre-morbid ongoing major depression or psychosis were additional exclusion criteria. Subjects were not allowed to participate in other training studies or perform physical therapy interventions targeting the lower limbs during the trial.

The iSCI subjects were recruited using the database of the University Hospital Balgrist, Zurich, Switzerland. Stroke subjects were recruited from the "Zentrum für ambulante Therapie ZAR", Zurich, Switzerland.

Six subjects (two SCI and four stroke subjects) were recruited for the trial (Table 1). However, two subjects dropped out of the trial after one and two weeks, respectively, because of personal reasons, so that only four subjects completed the trial.

**Table 1:** Characteristics of subjects recruited for the pilot trial (AIS - ASIA impairment scale [56], WISCI II - Walking Index for Spinal Cord Injury, version II [61,62])

**Subject**	**Age**	**Height (cm)**	**Weight (kg)**	**Sex**	**Medical diagnosis**	**Months p. injury**	**AIS**	**WISCI II**
P01	69	178	68	m	Incomplete tetraplegia sub C4, central cord syndrome syndrome	17	D	13
P02^*^	46	176	67	m	Ischemic stroke A. carotis interna left	19	n/a	18
P03	38	163	62	f	Ischemic stroke A. media left	12	n/a	20
P04	45	183	80	m	Ischemic stroke A. media left	8	n/a	15
P05^*^	48	168	65	m	Hemorrhagic stroke fronto-parieto-opercular	8	n/a	18
P06	69	178	80	m	Incomplete paraplegia sub Th8, stroke after tumor exspiration	13	D	12

^*^Subjects P02 and P05 dropped out of the study because of personal reasons before completing the four week training intervention.

### Training protocol

The subjects trained for 45 min (actual training time) four times a week during a period of four weeks, i.e. each subject performed 16 training sessions in total.

The first training session focused on the subject’s setup and adjustments within the device and was carried out by two therapists to reduce setup time. To allow subjects to acclimatize, training started with approximately 30% body-weight support and a treadmill speed of 1.9 km/h. All subjects initially started training using foot lifters (stroke subjects only at the affected side) to ensure foot clearance during swing phase. If control and strength of ankle dorsiflexion improved, the tension of the foot lifters was decreased until the point of volitional dorsiflexion was sufficient to remove the foot lifters.

In subsequent sessions, training intensity was increased progressively by changing walking speed, level of body-weight support and guidance force of the robot. The amount of BWS was adjusted individually in order for the subjects to achieve adequate knee extension during the stance phase and toe clearance during the swing phase. Adjustments were made according to the following priorities: 

1. The stiffness of the impedance controller (guidance force) was decreased as far as possible, until 0% guidance force was reached and the subject was training only with the Generalized Elastic Path Control strategy.

2. The tunnel width was increased from narrow as far as possible until the widest tunnel setting was reached.

3. Treadmill speed was increased as far as possible until the maximal speed of 4.0 km/h was reached.

4. Body-weight support was reduced as much as possible.

The adjustments listed above were made by the therapist continuously during each session. Based on experience in manual and robot-aided gait rehabilitation, the therapist subjectively judged gait quality and the subject’s level of motivation to decide if the subject was likely to tolerate a more challenging training. The goal for each training session was to reach the highest level of challenge, i.e. a guidance force of 0%, the widest tunnel setting, a treadmill speed of 4.0 km/h and the lowest level of body-weight support tolerated by the patient. Adaptations were always done systematically, following the order stated above.

If subjects were too exhausted to continue, which was judged by the therapist who subjectively assessed the quality of their walking patterns, training intensity was reduced by taking back the adjustments in reverse order. When subjects needed to be distracted from exhaustion towards the end of the 45 min of training, the visual feedback was changed to a coin-collecting game from the commercial "Lokomat System Augmented Feedback" [63].

When subjects were able to walk with the widest tunnel setting, we tested whether they were able to control the treadmill in Automatic Treadmill Speed Adaptation mode. If this was the case, 10 to 15 min of Automatic Treadmill Speed Adaptation were introduced in the training sessions to increase variability of the training and active participation of the subjects.

### Outcome measures and data recording

In the second training session (baseline), after two weeks of training and in the final training session after four weeks, we performed electromyography (EMG) of five leg muscles (rectus femoris (RF), vastus medialis (VM), biceps femoris (BF), tibialis anterior (TA) and gastrocnemius medialis (GM)) as well as heart rate measurements under five different training conditions: 

1. Walking on the treadmill with body-weight support but without the Lokomat (Free).

2. Walking with the Lokomat in Generalized Elastic Path Control mode (0% guidance force), with the maximal tunnel width (Wide).

3. Walking with the Lokomat in Generalized Elastic Path Control mode (0% guidance force), with the minimal tunnel width (Narrow).

4. Walking with the Lokomat in Generalized Elastic Path Control mode (0% guidance force), with the minimal tunnel width and an additional supportive flow of *k*_sup_ = 5Nm (Narrow^+^).

5. Walking with the Lokomat in position control mode, i.e. with the impedance controller set to 100% guidance force (Pos).

The level of body-weight support was set to one third of the subject’s body mass under all conditions, which proved to be sufficient for all subjects. The subjects walked for 2 min under each condition, while signals were recorded. After 90 s, they were questioned about their subjective feeling of effort (perceived exertion) using the Borg-Scale ranging from 6 to 20 [64,65]. Conditions were applied in randomized order, and patients were not informed about the order of the conditions.

EMG recordings were conducted according to the SENIAM guidelines [66]. In iSCI subjects, EMG signals were recorded from the weaker leg, which was determined after muscle testing according to the ASIA motor score [67]. In stroke subjects, the data was collected from the affected paretic leg. Signals were recorded with 1000 Hz and band-pass filtered between 30 to 300 Hz with an additional notch filter at 50 Hz.

The outcome measure to investigate the effects of training was the ten meter walking test (TMWT) [68], which assesses the time needed by the subject to ambulate 10 m. Both, self-selected velocity (SSV) and fast velocity (FV) of the subjects were determined, with the instruction to "walk at your normal, comfortable pace" and to "walk as fast as safely possible", respectively. The TMWT was performed once before the start of the trial and then again after every week of training, leading to a total of five measurements.

### Data analysis

#### EMG data

In order to classify the quantity and quality of muscle activity with different control strategies for the Lokomat, we computed an EMG metric according to [69]. This metric has been developed and validated for leg muscle activity during walking and assesses the similarity to a pattern of norm activity. To obtain patterns of norm activity for the muscles of interest, we recorded EMG data from 16 healthy subjects walking on a treadmill without the Lokomat (Table 2).

**Table 2:** Healthy subjects for obtaining EMG reference data

**Subject**	**Age**	**Height (cm)**	**Weight (kg)**	**Sex**
S01	24	169	66	f
S02	35	175	65	m
S03	24	178	73	f
S04	24	170	60	f
S05	23	172	66	f
S06	23	170	59	f
S07	27	171	58	f
S08	34	186	88	m
S09	19	163	49	f
S10	23	168	65	f
S11	31	185	85	m
S12	30	180	71	m
S13	25	175	65	m
S14	28	187	92	m
S15	24	183	80	m
S16	25	180	74	m

The recorded EMG signals were rectified and cut into single strides triggered by the heel strike signal of the force sensors of the treadmill. The single strides were normalized in time to 1000 samples each. All strides of a subject were then averaged. The EMG amplitudes were normalized to one, by dividing the signals by the maximal signal level recorded for each muscle. Finally, the average activity patterns of the single subjects were averaged to obtain the patterns of norm activity for each muscle.

**Figure 4:**
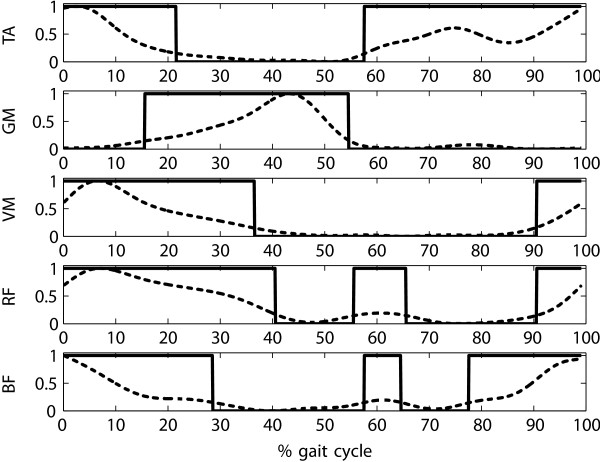
**Patterns of EMG norm activity over one gait cycle ranging from one initial contact (0% gait cycle) to the next initial contact (100% gait cycle).** The dashed line represents the average muscle activity recorded from healthy subjects S01–S16 during treadmill walking. The solid line represents the discrete "on"/"off" signal NormAct(*S*).

From the patterns of norm activity, binary on/off patterns were extracted by assigning the value "1" (representing "on") to phases above a defined threshold threshold_muscle_ and to "−1" (representing "off") for phases below threshold (Figure 4). The value for threshold_muscle_ was set to 15% of the maximum of the averaged muscle activity:

(1) $$\text{NormAct}(S)=\left\{\begin{array}{c} 1\;\;\text{EMG signal}>{\text{threshold}}_{\text{muscle}}\\ -1\;\;\text{EMG signal}\leq {\text{threshold}}_{\text{muscle}} \end{array}\right)$$

Averaged EMG signals of the subjects obtained under the different conditions mentioned above were then related to the norm activity patterns by two heuristics, one for the magnitude of the EMG signal, and one for the phase of the EMG signal [69]. The values obtained by the heuristics are confined to the interval [0,1]. Briefly summarized, the magnitude component yields a maximal value of 1 if the EMG signal has maximal amplitude during the "on" phases and zero amplitude during the "off" phases of the norm pattern. The phase component yields a maximal value of 1 if the EMG signal is always above threshold during the "on" phases and always below threshold during the "off" phases of the norm pattern. Magnitude components and phase components for all muscles are averaged to obtain one value which represents the similarity of overall muscle activity to the pattern of norm activity. Because of the simplified rectangular shape of the norm pattern, the amplitude component tends to yield values lower than 1 for physiological activity patterns. Based on exemplary data [69] and a validation experiment with a subset of the subjects listed in Table 2, we can expect EMG metric values of 0.7–0.8 for healthy subjects walking freely on a treadmill.

The EMG measurements were repeated three times (at baseline, after two weeks, and after four weeks). To obtain one representative value for each subject under each condition, we calculated the median of the EMG metric for these three separate measurements. As we were also interested in potential trends in the EMG metric over the course of the four weeks of training, we normalized the muscle activity of the subjects to the maximal EMG value of the average stride under the free walking condition during their first (baseline) measurement session.

To compare the EMG metrics obtained under the different conditions, we performed a Friedman test at the 5% significance level. In subsequent post-hoc tests, we applied the Bonferroni adjustment for multiple comparisons [70,71].

#### Physical effort

We normalized the heart rate values HR_during_ of the subjects during walking under the different Lokomat conditions to their heart rate HR_free_, which we recorded while they were walking on the treadmill without the Lokomat (condition Free):

(2) $${\text{HR}}_{\text{rel}}=\frac{{\text{Hr}}_{\text{during}}}{{\text{HR}}_{\text{free}}}.$$

As a measure of perceived physical effort, we asked subjects to rate their level of exertion on the Borg scale, which ranges from 6 to 20 [65,72], after 90 s of walking under the respective condition.

Analogous to the EMG metric, the median value of the three measurements (at baseline, after two weeks, and after four weeks) was calculated for relative heart rate HR_rel_ and Borg scale. Statistical comparisons of the different conditions were performed with a Friedman test at the 5% significance level and Bonferroni adjustment for multiple comparisons during post-hoc tests [70,71].

#### TMWT

To eliminate the effect of day to day fluctuations in the TMWT, we fit a linear model to the measured walking speeds of each subject:

(3) $$v_{\text{TMWT}}=\beta_{0}+\beta_{1}\times \frac{n_{\text{weeks}}}{4}.$$

In this model, *β*_0_corresponds to the initial walking speed of a subject at baseline, while *β*_1_ reflects the increase in walking speed after the four weeks of training in the pilot trial. The resulting model coefficients *β*_0_ and *β*_1_ were checked by t-tests (*α* = 0.05) for a significant difference to zero.

## Results

### Comparison of different training modes

**Figure 5:**
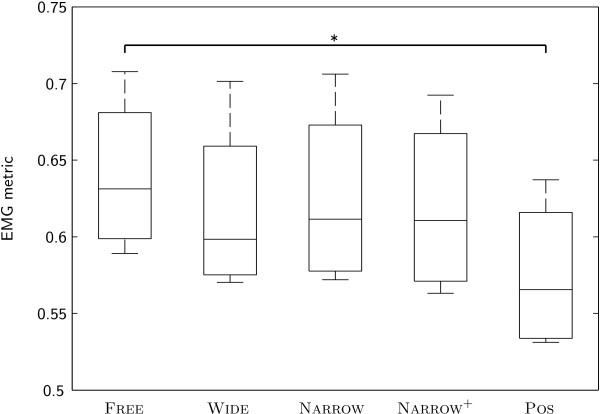
EMG metric of subjects under different conditions.

The EMG patterns of our subjects showed the highest similarity to the pattern of norm activity under condition Free, i.e. while they were walking on the treadmill without the Lokomat. The value of the EMG metric was significantly lower under condition Pos. There were no significant differences between other conditions (Figure 5).

**Figure 6:**
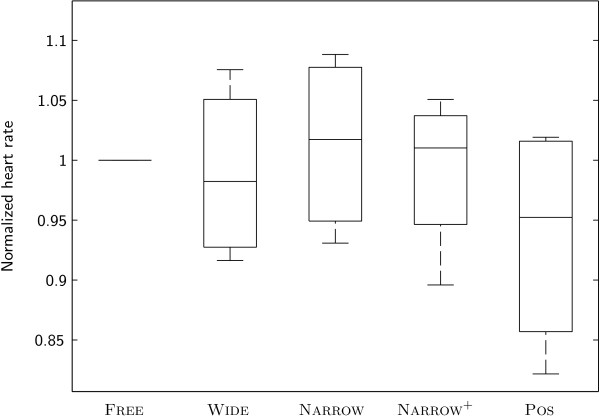
Heart rate of subjects under different conditions.

**Figure 7:**
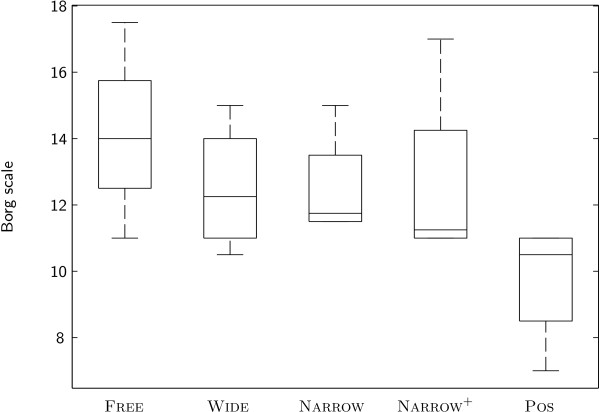
Borg scale of subjects under different conditions.

No significant differences between conditions were identified for relative heart rate (Figure 6) and Borg scale (Figure 7). However, for the Borg scale, a distinct trend towards reduced perceived exertion during condition Pos compared to the other conditions was visible.

The dashed line represents the average muscle activity recorded from healthy subjects S01–S16 during treadmill walking. The solid line represents the discrete "on"/"off" signal NormAct(*S*).

### TMWT

**Figure 8:**
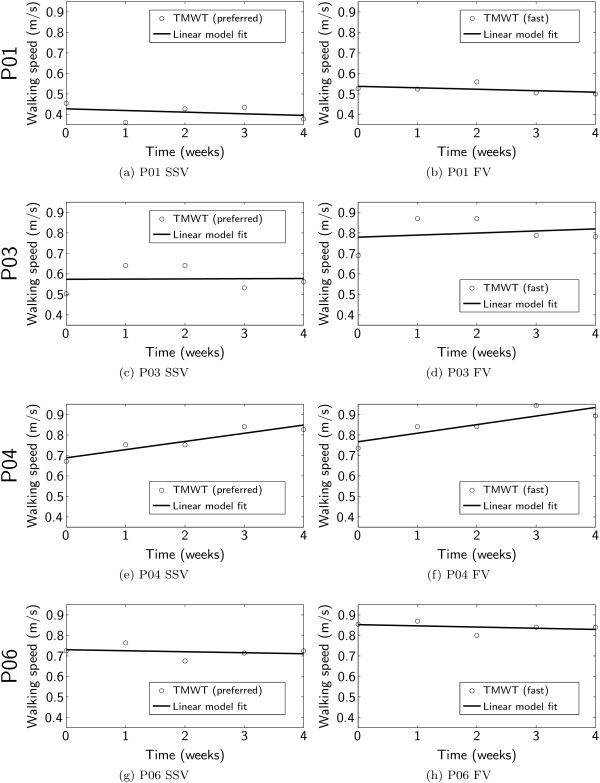
Results of ten meter walking test and regression model fit for each subject and for preferred, self-selected (SSV, left column) and fast (FV, right column) walking velocity.

In all iSCI subjects, the results of the TMWT fluctuated excessively from measurement to measurement (Figure 8).

Subject P04 showed a consistent improvement in walking speed over the four weeks of training, which is also reflected in the coefficients of the linear regression model fit (Table 3). The modeled increase in walking velocity during the training period was 0.16 m/s for the SSV and 0.167 m/s for the FV. The increase in SSV contributed significantly to the linear model (*p* = 0.024), while the increase in FV was distinct but just not significant (*p* = 0.065).

**Table 3:** Linear regression model coefficients of results in ten meter walking test

**Subject**	**TMWT type**	***β*_0_ (m/s)**	***p*(*β*_0_)**	***β***_**1**_ ( $\frac{m/s}{4\mathrm{weeks}}$)	***p*(*β*_1_)**
P01	SSV	**0.43**	0.001	-0.032	0.600
	FV	**0.54**	0.000	-0.028	0.405
P03	SSV	**0.57**	0.002	0.004	0.970
	FV	**0.78**	0.001	0.040	0.730
P04	SSV	**0.69**	0.000	**0.160**	0.024
	FV	**0.77**	0.000	0.167	0.065
P06	SSV	**0.73**	0.000	-0.020	0.688
	FV	**0.85**	0.000	-0.023	0.559

Significant model coefficients (*p* < 0.05) are marked in bold.

For subjects P01, P03, and P06, the modeled changes in walking speed were small ( $|\beta_{1}|\leq \frac{0.04\;\text{m/s}}{4\;\text{weeks}}$) and did not contribute significantly to the linear model (*p*(*β*_1_) ≥ 0.4).

## Discussion

### Feasibility of patient-cooperative robot-aided gait training

The patient-cooperative robot-aided gait training with the Generalized Elastic Path Control approach was tolerated well by all subjects and was performed without difficulties. One important aspect which contributed substantially to the feasibility was the combination of the Generalized Elastic Path Control approach with the impedance control approach. Especially during the first training sessions, subjects started with full guidance of the impedance controller, and the gradual decrease of guidance force allowed them to slowly get used to training with more active participation.

Despite using a fixed treadmill speed that was not directly influenced by the subjects, the freedom of timing in swing phase made it a constant challenge to keep the timing of walking synchronized with the virtual "ghost legs" of the avatar shown to the subjects. All patients quickly understood this task and mastered it with varying success during the single training sessions, typically with good initial performance which reduced when the level of challenge was increased, and when subjects got more exhausted.

In the subjective observation of the therapist, all subjects became more confident in influencing their gait pattern with the Lokomat over the four weeks of training.

### Short-term reaction to patient-cooperative vs. non-cooperative training

Our second research question aimed at identifying the different short-term reactions of patients to different control strategies for the Lokomat, and more specifically, to patient-cooperative vs. non-cooperative control strategies.

Despite the limited statistical power associated with our small number of subjects of *n* = 4, our results clearly show that the non-cooperative position control mode causes subjects to activate their leg muscles in a way that is significantly less similar to a physiological activation pattern than when they are walking on a treadmill without the Lokomat (Figure 5). These findings are consistent with earlier studies by other groups investigating the influence of (non-cooperative) robot-aided gait training with the Lokomat [73,74]. In contrast, muscle activity under all patient-cooperative Lokomat conditions was more similar to the norm activation pattern than under the non-cooperative condition.

Interestingly, the effects of the different control modes on muscle activity are well in line with the trend visible in the results of the Borg scale rating of the perceived exertion of the subjects (Figure 7). The human motor system uses every possibility to "slack" [75], and it seems that the additional pressure of the challenging rehabilitation training causes subjects to relieve this pressure by relying strongly on the unconditional support of the non-cooperative position control mode. The patient-cooperative modes (independent of tunnel width or the presence of an additional flow of support) appear to keep subjects sufficiently active to exhibit near-to-normal muscle activity and perceived exertion.

These findings extend the results reported in [51], which only had investigated the effects of patient-cooperative robot-aided training on the *quantity* of muscle activity, by showing that the patient-cooperative control modes also improve the *quality* of the patterns of muscle activity. Impedance control [38] with medium controller stiffness did not show significantly increased levels of muscle activity in this previous investigation [51], whereas impedance control with low controller stiffness has not been practical with the Lokomat, as the effects of inertia cause perturbations and result in a non-physiological gait pattern. As a consequence, we focused the present investigations on the patient-cooperative Path Control approach that improves the transparency of the device so that natural walking with more freedom becomes possible.

Thus, the patient-cooperative control mode investigated in the study allows patients to train more actively and—as a consequence—with more physiological muscle activity than the established non-cooperative position control mode. Patients are aware of their increased activity, a fact which could contribute positively to their motivation.

### Effect of training on walking function

The last research question addressed the effect of four weeks of patient-cooperative robot-aided gait training on walking function. More specifically, we were interested in changes in walking speed in the TMWT. Only one subject (P04) showed a significant change in this outcome measure, which increased by 0.16 m/s (SSV).

In comparison to results obtained by other studies (Table 4), this improvement distinctly exceeds the gains in walking speed of 0.05–0.07 m/s typically achieved in four weeks of non-cooperative Lokomat training.

**Table 4:** Improvements in walking speed (SSV) in clinical trials evaluating robot-aided gait training with the Lokomat

	**4 weeks**		**8 weeks**	
**Clinical trial**	**RT**	**MT**	**RT**	**MT**
Wirz et al. [76]	0.055^*^	–	0.11±0.1	–
Husemann et al. [29]	0.05±0.05	0.08±0.03	–	–
Hornby et al. [34]	0.07±0.07	0.13±0.11	–	–
Hidler et al. [33]	0.06±0.03	0.18±0.03	0.12±0.03	0.25±0.03

Numbers report increases in SSV in m/s with respect to baseline after 4 or 8 weeks of training. Standard deviations are listed to the right of the "±" sign. RT: robot-aided training with the Lokomat, MT: manual training.

^*^The result after 4 weeks for the trial by Wirz et al. [76] is interpolated based on the reported result after 8 weeks, assuming a linear increase of walking speed over the course of therapy.

Subjects P01, P03, and P06 did not significantly change their overground walking speed, even though the therapists subjectively reported that the subjects visibly improved their gait in the Lokomat. P01 and P06 were older than the other subjects (both 69 years old) and did not seem to be sufficiently flexible in their behavior to modify their accustomed compensatory walking strategies for daily life. Subject P03 showed a remarkable improvement during the first two weeks of training, but fell back during the second two weeks (Figures 8c and 8d).

The limited number of subjects in this pilot trial does not permit valid conclusions on the effect of patient-cooperative robot-aided gait training on walking function. With respect to a potential larger randomized controlled trial, several aspects should be considered: Even though chronic subjects are attractive for pilot trials because all improvement can be attributed to the intervention, it is questionable whether they constitute a meaningful population for assessing the realistic potential of a specific rehabilitation intervention.

As it very likely happened in two of the subjects in this pilot trial, improvements may just not take place if established compensation strategies are already so deeply engrained that an additional behavioral intervention would be necessary in addition to the motor training. Therefore, we need to start understanding why specific patients respond to certain interventions and others don’t in order to tailor robot-aided therapy much more to their needs. Modeling recovery based on data from larger trials as e.g. done with data from the EXCITE trial for upper extremity stroke rehabilitation [77] may be an important first step in this direction.

Moreover, to obtain a better understanding of the specific effects of the training of single subjects in detail, and be ultimately able to link the success or failure of specific interventions to the precise nature of specific deficits, data acquisition during the trials should be extended to include more parameters such as step lengths, detailed ground reaction forces, and ideally even trunk movements.

Thus, larger clinical trials investigating the effects of robot-aided therapy interventions should focus rather on acute and subacute patient populations and tailor the interventions to the specific needs and deficits that an individual patient needs to overcome in order to reach the threshold of function required for increasing the use of the affected limb in everyday life [78].

## Conclusion

Patient-cooperative, robot-aided gait training with stroke and iSCI patients is feasible—not only for single training sessions, as demonstrated previously [51], but also over the course of a realistic training period of four weeks. Patients do not only participate more actively than during non-cooperative, position-controlled robot-aided gait training, they also show more physiological patterns of muscle activity in their main leg muscles.

Thus, patient-cooperative robot-aided gait training overcomes the main points of criticism against robot-aided gait training: It enables patients to train walking in an active, variable and more natural way. However, the question remains open how much more effective robot-aided gait training may become due to these improvements. One of the chronic subjects in this trial showed very encouraging results, whereas the others were not receptive to the therapy. In this respect, an important topic of future rehabilitation research should be the question what makes certain patients respond to a specific rehabilitation intervention while others don’t.

A large, possibly multi-center randomized controlled clinical trial is required to shed more light on this question. In contrast to previous trials, robot-aided training tasks should be systematically tailored to the specific needs of the individual patients, based on e.g. computational models of patient recovery driven by data from clinical and robot-aided assessment. Distributing the proposed patient-cooperative control algorithms to a substantial fraction of the more than 300 Lokomat robots in clinical use world-wide may provide an important basis for such a trial.

## Endnotes

^a^http://www.rehatechnologies.eu

## Competing interests

AD is employed by Hocoma AG, Volketswil, Switzerland. Hocoma is the manufacturer of the Lokomat gait rehabilitation robot, which was used as the platform to implement the patient-cooperative control algorithm for robot-aided gait training investigated in this study. AS, RL, HV, and RR declare that they have no competing interests.

## Authors’ contributions

AS performed the training and measurements of all subjects, study design and coordination, data analysis, statistical analysis, and drafted the manuscript. RL contributed to the recruitment of subjects and assisted in the measurements and training sessions. HV contributed to the design and implementation of the patient-cooperative control algorithm. RR participated in the design and coordination of the study and assisted with drafting the manuscript. AD contributed to the design and implementation of the patient-cooperative control algorithm, the design and coordination of the study, and assisted with data analysis, statistical analysis and with drafting the manuscript. All authors read and approved the final manuscript.

## References

[B1] Lennon S The Bobath concept: a critical review of the theoretical assumptions that guide physiotherapy practice in stroke rehabilitation Phys Therapy Rev 1996 35 45

[B2] Seligman MEP Helplessness: On Depression, Development, and Death 1975 San Francisco: WH Freeman

[B3] Taub E Miller NE Novack TA Cook EW Fleming WC Nepomuceno CS Connell JS Crago JE Technique to improve chronic motor deficit after stroke Arch Phys Med Rehabilitation 1993 74 4 347 354 8466415

[B4] Wolf SL Winstein CJ Miller JP Taub E Uswatte G Morris D Giuliani C Light KE Nichols-Larsen D for the EXCITE Investigators Effect of constraint-induced movement therapy on upper extremity function 3 to 9 months after stroke: the EXCITE randomized clinical trial JAMA 2006 296 17 2095 2104 10.1001/jama.296.17.2095 17077374

[B5] Wolf SL Winstein CJ Miller JP Thompson PA Taub E Uswatte G Morris D Blanton S Nichols-Larsen D Clark PC Retention of upper limb function in stroke survivors who have received constraint-induced movement therapy: the EXCITE randomised trial Lancet Neurol 2008 7 1 33 40 10.1016/S1474-4422(07)70294-6 18077218 PMC2329576

[B6] Lin K-C Wu C-Y Liu J-S Chen Y-T Hsu C-J Constraint-induced therapy versus dose-matched control intervention to improve motor ability, basic/extended daily functions, and quality of life in stroke Neurorehabil Neural Repair 2009 23 2 160 165 18981188 10.1177/1545968308320642

[B7] Maier IC Baumann K Thallmair M Weinmann O Scholl J Schwab ME Constraint-induced movement therapy in the adult rat after unilateral corticospinal tract injury J Neurosci 2008 28 38 9386 9403 10.1523/JNEUROSCI.1697-08.2008 18799672 PMC6671131

[B8] Barbeau H Wainberg M Finch L Description and application of a system for locomotor rehabilitation Med Biol Eng Comput 1987 25 3 341 344 10.1007/BF02447435 3449731

[B9] Barbeau H Visintin M Optimal outcomes obtained with body-Weight support combined with treadmill training in stroke subjects Arch Phys Med Rehabilitation 2003 84 10 1458 1465 10.1016/S0003-9993(03)00361-7 14586912

[B10] Barbeau H Nadeau S Garneau C Physical determinants, emerging concepts, and training approaches in gait of individuals with spinal cord injury J Neurotrauma 2006 23 3-4 571 585 10.1089/neu.2006.23.571 16629638

[B11] Cai LL Fong AJ Otoshi CK Liang Y Burdick JW Roy RR Edgerton VR Implications of assist-as-needed robotic step training after a complete spinal cord injury on intrinsic strategies of motor learning J Neurosci 2006 26 41 10564 10568 10.1523/JNEUROSCI.2266-06.2006 17035542 PMC6674681

[B12] Dromerick A Lum P Hidler J Activity-based therapies NeuroRX 2006 3 4 428 438 10.1016/j.nurx.2006.07.004 17012056 PMC3593413

[B13] Norman K Pepin A Ladouceur M Barbeau H A treadmill apparatus and harness support for evaluation and rehabilitation of gait Arch Phys Med Rehabilitation 1995 76 8 772 778 10.1016/S0003-9993(95)80533-8 7632134

[B14] Visintin M Barbeau H Korner-Bitensky N Mayo NE A new approach to retrain gait in stroke patients through body weight support and treadmill stimulation Stroke; J Cerebral Circulation 1998 29 6 1122 1128 10.1161/01.STR.29.6.1122 9626282

[B15] Dobkin B Apple D Barbeau H Basso M Behrman A Deforge D Ditunno J Dudley G Elashoff R Fugate L Harkema S Saulino M Scott M Weight-supported treadmill vs over-ground training for walking after acute incomplete SCI Neurology 2006 66 4 484 493 10.1212/01.wnl.0000202600.72018.39 16505299 PMC4102098

[B16] van Hedel Hubertus JA Weight-supported treadmill versus over-ground training after spinal cord injury: from a physical therapist’s point of view PHYS THER 2006 86 10 1444 1447 10.2522/ptj.2006.86.10.1444 17012649

[B17] Sullivan KJ Brown DA Klassen T Mulroy S Ge T Azen SP Winstein CJ Effects of task-specific locomotor and strength training in adults who were ambulatory after stroke: results of the STEPS randomized clinical trial Phys Therapy 2007 87 12 1580 1602 10.2522/ptj.20060310 17895349

[B18] Colombo G Joerg M Schreier R Dietz V Treadmill training of paraplegic patients using a robotic orthosis J Rehabil Res Dev 2000 37 6 693 700 11321005

[B19] Riener R Lünenburger L Maier I Colombo G Dietz V Locomotor training in subjects with sensori-motor deficits: an overview of the robotic gait orthosis Lokomat J Healthcare Eng 2010 1 2 l216

[B20] Hesse S Uhlenbrock D A mechanized gait trainer for restoration of gait J Rehabilitation Res Dev 2000 37 6 701 708 11321006

[B21] Sayers SP Krug J Robotic gait-assisted therapy in patients with neurological injury Missouri Med 2008 105 2 153 158 18453195

[B22] Schmidt H Werner C Bernhardt R Hesse S Krüger Jörg Gait rehabilitation machines based on programmable footplates J Neuroengineering Rehabilitation 2007 4 2+ 10.1186/1743-0003-4-2 PMC1804273 17291335

[B23] Veneman JF Kruidhof R Hekman EEG Ekkelenkamp R Van Asseldonk EHF van der Kooij H Design and evaluation of the LOPES exoskeleton robot for interactive gait rehabilitation IEEE Trans Neural Syst Rehabil Eng 2007 15 3 379 386 17894270 10.1109/tnsre.2007.903919

[B24] Banala SK Agrawal SK Scholz JP Active Leg Exoskeleton (ALEX) for gait rehabilitation of motor-impaired patients Proc IEEE 10th Int Conf Rehabil Robot 2007 Noordwijk 401 407

[B25] Aoyagi D Ichinose WE Harkema SJ Reinkensmeyer DJ Bobrow JE A robot and control algorithm that can synchronously assist in naturalistic motion during body-weight-supported gait training following neurologic injury IEEE Trans Neural Syst Rehabil Eng 2007 15 3 387 400 17894271 10.1109/TNSRE.2007.903922

[B26] Dollar AM Herr H Lower extremity exoskeletons and active orthoses: challenges and state-of-the-art Robotics IEEE Trans on 2008 24 1 144 158

[B27] Mehrholz J Werner C Kugler J Pohl M Electromechanical-assisted training for walking after stroke Cochrane Database of Systematic Rev (Online) 2007 4 10.1002/14651858.CD006185.pub2 17943893

[B28] Mehrholz J Kugler J Pohl M Locomotor training for walking after spinal cord injury Cochrane Database of Systematic Rev (Online) 2008 2 10.1002/14651858.CD006676.pub2 18425962

[B29] Husemann B Müller F Krewer C Heller S Koenig E Effects of locomotion training with assistance of a robot-driven gait orthosis in hemiparetic patients after stroke: a randomized controlled pilot study Stroke; J Cerebral Circulation 2007 38 2 349 354 10.1161/01.STR.0000254607.48765.cb 17204680

[B30] Mayr A Kofler M Quirbach E Matzak H Frohlich K Saltuari L Prospective, blinded, randomized crossover study of gait rehabilitation in stroke patients using the lokomat gait orthosis Neurorehabil Neural Repair 2007 21 4 307 314 10.1177/1545968307300697 17476001

[B31] Schwartz I Sajin A Fisher I Neeb M Shochina M Katz-Leurer M Meiner Z The effectiveness of locomotor therapy using robotic-assisted gait training in subacute stroke patients: a randomized controlled trial PM R : J Injury, Funct, Rehabilitation 2009 1 6 516 523 10.1016/j.pmrj.2009.03.009 19627940

[B32] Westlake Kelly Patten C Pilot study of Lokomat versus manual-assisted treadmill training for locomotor recovery post-stroke J NeuroEngineering Rehabilitation 2009 6 1 18+ 10.1186/1743-0003-6-18 PMC2708184 19523207

[B33] Hidler J Nichols D Pelliccio M Brady K Campbell DD Kahn JH Hornby GT Multicenter randomized clinical trial evaluating the effectiveness of the Lokomat in subacute stroke Neurorehabil Neural Repair 2009 23 1 5 13 19109447 10.1177/1545968308326632

[B34] Hornby GT Campbell DD Kahn JH Demott T Moore JL Roth HR Enhanced gait-related improvements after therapist- versus robotic-assisted locomotor training in subjects with chronic stroke: a randomized controlled study Stroke 2008 39 6 1786 1792 10.1161/STROKEAHA.107.504779 18467648

[B35] Murray AF Edwards PJ Synaptic weight noise during MLP learning enhances fault-tolerance, generalisation and learning trajectory IEEE Trans on Neural Networks 1993 5 5 792 802 10.1109/72.317730 18267852

[B36] Bernstein NA The Co-ordination and regulation of movements 1967 First English edition. Pergamon Press Ltd.

[B37] Hogan N Impedance control - an approach to manipulation. I - Theory. II - Implementation. III - Applications ASME Trans J Dynamic Syst Meas Control B 1985 107 1 24 10.1115/1.3140702

[B38] Riener R Lünenburger L Jezernik S Anderschitz M Colombo G Dietz V Patient-cooperative strategies for robot-aided treadmill training: first experimental results IEEE Trans Neural Syst Rehabil Eng 2005 13 3 380 394 10.1109/TNSRE.2005.848628 16200761

[B39] Emken JL Bobrow JE Reinkensmeyer DJ Robotic movement training as an optimization problem: designing a controller that assists only as needed IEEE Int. Conf. on Rehabilitation Robotics (ICORR) 2005 Chicago 307 312

[B40] Reinkensmeyer DJ Emken JL Cramer SC Robotics, motor learning, and neurologic recovery Ann Rev Biomed Eng 2004 6 497 525 10.1146/annurev.bioeng.6.040803.140223 15255778

[B41] Riener R Fuhr T Patient-driven control of FES-supported standing up: a simulation study Rehabilitation Eng, IEEE Trans on 1998 6 2 113 124 10.1109/86.681177 9631319

[B42] Jezernik S Colombo G Morari M Automatic gait-pattern adaptation algorithms for rehabilitation with a 4-DOF robotic orthosis IEEE Trans Robot Autom 2004 20 3 574 582 10.1109/TRA.2004.825515

[B43] Riener R Frey M Bernhardt M Nef T Colombo G Human-centered rehabilitation robotics Rehabilitation Robotics, 2005 2005 319 322

[B44] Reinkensmeyer DJ Aoyagi D Emken JL Galvez JA Ichinose W Kerdanyan G Maneekobkunwong S Minakata K Nessler JA Weber R Roy RR de Leon R Bobrow JE Harkema SJ Edgerton VR Tools for understanding and optimizing robotic gait training J Rehabil Res Dev 2006 43 5 657 670 10.1682/JRRD.2005.04.0073 17123206

[B45] van Asseldonk EHF Veneman JF Ekkelenkamp R Buurke JH van der Helm FCT van der Kooij H The effects on kinematics and muscle activity of walking in a robotic gait trainer during zero-force control IEEE Trans Neural Syst Rehabil Eng 2008 16 4 360 370 18713676 10.1109/TNSRE.2008.925074

[B46] Vallery H Guidali M Duschau-Wicke A Riener R Dössel Olaf, Schlegel WolfgangC. Patient-cooperative control: providing safe support without restricting movement World Congress on Medical Physics and Biomedical Engineering, September 7 - 12, 2009, Munich, Germany 2009 Berlin, Heidelberg: Springer Berlin Heidelberg 166 169

[B47] Emken JL Harkema SJ Beres-Jones JA Ferreira CK Reinkensmeyer DJ Feasibility of manual teach-and-replay and continuous impedance shaping for robotic locomotor training following spinal cord injury IEEE Trans Biomed Eng 2008 55 1 322 334 18232376 10.1109/TBME.2007.910683

[B48] Marchal Crespo L Reinkensmeyer D Review of control strategies for robotic movement training after neurologic injury J Neuroengineering rehabilitation 2009 6 1 20+ 10.1186/1743-0003-6-20 PMC2710333 19531254

[B49] Cai LL Fong AJ Otoshi CK Liang YQ Cham JG Zhong H Roy RR Edgerton VR Burdick JW Effects of consistency vs. variability in robotically controlled training of stepping in adult spinal mice. 2005 Chicago

[B50] Duschau-Wicke A von Zitzewitz J Caprez A Lünenburger L Riener R Path control: a method for patient-cooperative robot-aided gait rehabilitation IEEE Trans Neural Syst Rehabilitation Eng 2010 18 1 38 48 10.1109/TNSRE.2009.2033061 20194054

[B51] Duschau-Wicke A Caprez A Riener R Patient-cooperative control increases active participation of individuals with SCI during robot-aided gait training J NeuroEngineering Rehabilitation 2010 7 1 43+ 10.1186/1743-0003-7-43 PMC2949707 20828422

[B52] Frey M Colombo G Vaglio M Bucher R Jörg M Riener R A novel mechatronic body weight support system IEEE Trans Neural Syst Rehabil Eng 2006 14 3 311 321 10.1109/TNSRE.2006.881556 17009491

[B53] Vallery H Duschau-Wicke A Riener R Generalized elasticities improve patient-cooperative control of rehabilitation robots IEEE Int Conf on Rehabilitation Robotics (ICORR) 2009 535 541

[B54] Vallery H Duschau-Wicke A Riener R Optimized passive dynamics improve transparency of haptic devices IEEE Int Conf Robot Aut (ICRA) 2009 301 306

[B55] von Zitzewitz J Bernhardt M Riener R A Novel method for automatic treadmill speed adaptation IEEE Trans Neural Syst Rehabil Eng 2007 15 3 401 409 17894272 10.1109/TNSRE.2007.903926

[B56] Maynard FM Bracken MB Creasey G Ditunno JF Donovan WH Ducker TB Garber SL Marino RJ Stover SL Tator CH Others International standards for neurological and functional classification of spinal cord injury Spinal Cord 1997 35 266 274 10.1038/sj.sc.3100432 9160449

[B57] Itzkovich M Gelernter I Biering-Sorensen F Weeks C Laramee MT Craven BC Tonack M Hitzig SL Glaser E Zeilig G Aito S Scivoletto G Mecci M Chadwick RJ Masry WS Osman A Glass CA Silva P Soni BM Gardner BP Savic G Bergström EM Bluvshtein V Ronen J Catz A The Spinal Cord Independence Measure (SCIM) version III: reliability and validity in a multi-center international study Disabil Rehabil 2007 29 24 1926 1933 10.1080/09638280601046302 17852230

[B58] Cuthbert SC Goodheart GJ On the reliability and validity of manual muscle testing: a literature review Chiropractic Osteopathy 2007 15 4+ 10.1186/1746-1340-15-4 17341308 PMC1847521

[B59] Schmitt WH Cuthbert SC Common errors and clinical guidelines for manual muscle testing: "the arm test" and other inaccurate procedures Chiropractic Osteopathy 2008 16 16+ 10.1186/1746-1340-16-16 19099575 PMC2628341

[B60] Cockrell JR Folstein MF Mini-mental state examination Principles Pract Geriatric Psychiatry 1988 140 141

[B61] Dittuno PL Dittuno JF Walking index for spinal cord injury (WISCI II): scale revision Spinal Cord 2001 39 654 656 10.1038/sj.sc.3101223 11781863

[B62] van Hedel HJ Dietz V European multicenter study on human spinal cord injury EM-SCI study group Walking during daily life can be validly and responsively assessed in subjects with a spinal cord injury Neurorehabilitation Neural Repair 2009 23 2 117 124 18997156 10.1177/1545968308320640

[B63] Zimmerli L Duschau-Wicke A Mayr A Riener R Lünenburger L Virtual reality and gait rehabilitation Augmented feedback for the Lokomat Virtual Rehabilitation International Conference 2009 150 153

[B64] Borg G Perceived exertion as an indicator of somatic stress Scand J Rehabilitation Med 1970 2 2 92 98 5523831

[B65] Lewis JE Nash MS Hamm LF Martins SC Groah SL The relationship between perceived exertion and physiologic indicators of stress during graded arm exercise in persons with spinal cord injuries Arch Phys Med Rehabilitation 2007 88 9 1205 1211 10.1016/j.apmr.2007.05.016 17826469

[B66] Hermens HJ Freriks B Merletti R Stegeman D Blok J Rau G Disselhorst-Klug C Haegg G European Recommendations for Surface Electromyography 1999

[B67] Marino RJ Barros T Biering-Sorensen F Burns SP Donovan WH Graves DE Haak M Hudson LM Priebe MM ASIA Neurological Standards Committee 2002 International standards for neurological classification of spinal cord injury J Spinal Cord Med 2003 26 Suppl 1 10.1080/10790268.2003.11754575 16296564

[B68] Alexander MS Anderson KD Biering-Sorensen F Blight AR Brannon R Bryce TN Creasey G Catz A Curt A Donovan W Ditunno J Ellaway P Finnerup NB Graves DE Haynes BA Heinemann AW Jackson AB Johnston MV Kalpakjian CZ Kleitman N Krassioukov A Krogh K Lammertse D Magasi S Mulcahey MJ Schurch B Sherwood A Steeves JD Stiens S Tulsky DS van Hedel HJA Whiteneck G Outcome measures in spinal cord injury: recent assessments and recommendations for future directions Spinal Cord 2009 47 8 582 591 10.1038/sc.2009.18 19381157 PMC2722687

[B69] Ricamato AL Hidler JM Quantification of the dynamic properties of EMG patterns during gait J Electromyography Kinesiology 2005 15 4 384 392 10.1016/j.jelekin.2004.10.003 15811609

[B70] Gibbons JD Nonparametric Statistical Inference 1985 Marcel Dekker Ltd

[B71] Hochberg Yosef Tamhane AjitC Multiple Comparison Procedures (Wiley Series in Probability and Statistics) 1987 Wiley

[B72] Goosey-Tolfrey V Lenton J Goddard J Oldfield V Tolfrey K Eston R Regulating intensity using perceived exertion in spinal cord-injured participants Med Sci Sports Exercise 2010 42 3 608 613 10.1249/MSS.0b013e3181b72cbc 19952816

[B73] Hidler JM Wall AE Alterations in muscle activation patterns during robotic-assisted walking Clin Biomech 2005 20 2 184 193 10.1016/j.clinbiomech.2004.09.016 15621324

[B74] Israel JF Campbell DD Kahn JH Hornby GT Metabolic costs and muscle activity patterns during robotic- and therapist-assisted treadmill walking in individuals with incomplete spinal cord injury Phys Therapy 2006 86 11 1466 1478 10.2522/ptj.20050266 17079746

[B75] Reinkensmeyer DJ Maier MA Guigon E Chan V Akoner O Wolbrecht ET Cramer SC Bobrow JE Do robotic and non-robotic arm movement training drive motor recovery after stroke by a common neural mechanism? Experimental evidence and a computational model Conf 2009 2439 2441 10.1109/IEMBS.2009.5335353 19965205

[B76] Wirz M Zemon DH Rupp R Scheel A Colombo G Dietz V Hornby TG Effectiveness of automated locomotor training in patients with chronic incomplete spinal cord injury: a multicenter trial Arch Phys Med Rehabil 2005 86 4 672 680 10.1016/j.apmr.2004.08.004 15827916

[B77] Schweighofer N Han CE Wolf SL Arbib MA Winstein CJ A functional threshold for long-term use of hand and arm function can be determined: predictions from a computational model and supporting data from the Extremity Constraint-Induced Therapy Evaluation (EXCITE) Trial Phys therapy 2009 89 12 1327 1336 10.2522/ptj.20080402 PMC2794477 19797304

[B78] Han CE Arbib MA Schweighofer N Stroke rehabilitation reaches a threshold PLoS Computat Biol 2008 4 8 10.1371/journal.pcbi.1000133 PMC2527783 18769588

